# Parental knowledge, awareness and acceptance of silver diamine treatment among patients from Modinagar, Uttar Pradesh, India

**DOI:** 10.6026/97320630018553

**Published:** 2022-06-30

**Authors:** Zohra Jabin, Iffat Nasim, V Vishnu Priya

**Affiliations:** 1 Saveetha Dental College, Saveetha Institute of Medical and Technical Sciences, Saveetha University, Chennai, India

**Keywords:** Silver diamine fluoride, knowledge, awareness, acceptance, dental caries

## Abstract

Silver diamine fluoride has emerged as a non invasive effective therapy for arresting active carious lesions. Therefore, it is of interest to assess parental knowledge, awareness and acceptance of SDF. A cross sectional questionnaire based survey was conducted among 62 parents visiting the Department of Pedodontics along with their children in Modinagar. A 15 item pre-validated questionnaire was used to record baseline characteristics of child's parents and their knowledge, awareness and acceptance of SDF for their child for prevention of dental caries. 80.6% participants were aware that SDF is painless procedure whereas majority of the participants were not sure about whether placement of filling required post SDF treatment (48.4%), SDF treatment be done outside dental clinic (61.3%), and about any agents that reduce SDF staining (64.5%). Majority 71% participants were aware about SDF treatment's side effects, 90.3% aware whether their child experienced teeth blackening after SDF treatment and 83.9% aware about whether SDF treatment immediately relieves the child's pain. 90.3% preferred SDF treatment, 83.9% opted for SDF treatment despite of its tooth blackening effects. A statistically significant association was found between SDF acceptance and age of their child. There is a lack of knowledge about SDF among the study participants emerging a need for proper education to them when they visit a dental setting with their child. Although it causes discolouration of teeth post SDF treatment, still parents are willing to accept SDF as a treatment modality for their child's oral health.

## Background:

Early childhood caries (ECC) continue to affect the vast majority of children around the world, especially in the developing world. In India, the prevalence of caries in 5 years old was reported around 50% whereas a recent meta-analysis calculated the pooled prevalence of ECC is 40% among Indian children [1, 2 and 3]. This data shows ECC continues to persist as a public health challenge in India and untreated cases of dental caries in young children continue to be an extensive challenge for oral health care professionals and policymakers. There is a strong evidence and literature support regarding the clinical effectiveness of topically applied fluorides. They are efficacious in both prevention against caries attack and inhibition of virulent bacteria. [4, 5, 6] Silver diamine fluoride has emerged as a noninvasive effective therapy for arresting active carious lesions and preventing occurrence of new caries by exerting its re-mineralizing effects. Its non surgical approach along with ease of use makes it desirable, especially for developing low income countries where population encounter low accessibility and utilization of basic dental procedures and struggle to combat the economic burden placed on them due to high costs of caries treatment. Its cost-effectiveness, and simple application procedure also advocates its use as an appropriate intervention for community settings. [7, 8] SDF has the potential to address the epidemic of untreated early childhood caries in young children especially in populations where surgical management of decay is not an option. [9,10].

Despite its known benefits and evidence to support its efficacy, usage of SDF is still not frequent in clinical dentistry. The major reason is the permanent dark staining SDF leaves after the application. This causes aesthetic concerns and discontent of parents, restricted socializing by children which negatively impacts their oral health-related quality of life. [11] This visible feature of appearance may not be acceptable for some children and parents and hence, it is necessary to inform them of this outcome of SDF therapy, during the Pre-treatment discussions for the assurance and satisfaction. [12] Few studies had tried to gain an insight about the parental understanding and their acceptance regarding the aesthetics after SDF application. Most of these studies had been conducted in developed countries except one. [12,13,14, 15] Therefore we conducted an exploratory questionnaire survey in a dental hospital of Modinagar to assess the parental knowledge, awareness and acceptance of SDF as a caries management method for their child. The association of parents' acceptability with factors such as the location of teeth, child's behaviour, age, gender or socioeconomic status was assessed.

## Methodology:

A cross sectional questionnaire based survey was conducted among 62 parents visiting the Department of Pedodontics along with their children in Modinagar. Parents whose children were between 3-14 years of age and was priorly treated with SDF was invited to participate in their subsequent appointment or contacted through online mode for this study. Ethical clearance was obtained from the institutional ethical committee before data collection. Written informed consent of parent was obtained from each study participant. All guidelines laid down by "Declaration of Helsinki" were followed in conducting the study due to sensitiveness of the study. Parents who were willing to participate were included in the study. A 15 item self formulated and pre-validated questionnaire was used to record baseline characteristics of the child's parents and their knowledge, awareness and acceptance of SDF for their child for the prevention of dental caries. We tested the questionnaire for clarity, ease of completion and timing and modified it according to parental comments. The questionnaire was also translated to Hindi and a second bilingual paediatric dental care provider reviewed it to ensure the identical intent of each question. Most parents completed the surveys on a tablet. A printed survey questionnaire was distributed to parents who were uncomfortable with using a tablet computer and recorded their answers electronically. Data were entered into Microsoft Excel and differences between the groups were checked using SPSS (Statistical Package for Social Sciences) Version 16.0; IBM SPSS Inc., Chicago, IL, USA. The data were subjected to quantitative analysis. Chi-square test was used to test the significant difference between the three groups of professionals (P ≤ 0.05). The normality of the data was assessed using Shapiro wilk test. The level of significance and confidence interval were 5% and 95% respectively.

## Results:

The present short-term study with limited samples demonstrates the usefulness of SDF in arresting active caries lesion of children aged 3-14 years and suggested that SDF is a suitable treatment option. Figure 1(see PDF) depicts that a majority 41.9% of the participants were in the age group of 31-35 years followed by 21% who were above 40 years. Figure 2(see PDF) describes that a majority 32.3% of the children were in the age group of 3-5 years followed by 22.6% who were in the age group 6-8 years. A majority of 61.3% of the participants were males whereas 38.7% of them were females which are described in Figure 3(see PDF). Since the study participants belonged to the affluent background, about half of the study participants had a higher degree that is post-graduation whereas only 19.4% were undergraduates which is depicted in Figure 4(see PDF). Table 1(see PDF) describes the distribution of responses of the study participant's knowledge, awareness and acceptance of Silver Diamine treatment. On assessing the knowledge based questions, it was seen that 80.6% participants were aware that SDF is a painless procedure whereas majority of the participants were not sure about whether placement of filling required post SDF treatment (48.4%), SDF treatment be done outside dental clinic (61.3%), and about any agents that reduce SDF staining (64.5%). On assessing the awareness based questions, it was seen that a majority 71% participants were aware about SDF treatment's side effects, 90.3% aware whether their child experienced teeth blackening after SDF treatment and 83.9% aware about whether SDF treatment immediately relieves the child's pain. 90.3% preferred SDF treatment, 83.9% opted for SDF treatment in spite of its tooth blackening effects. Table 2(see PDF) shows the association of acceptance of SDF use among the study participants with their education, gender and their age of child. A statistically significant association was found between SDF acceptance and age of the child. Although SDF has a drawback of staining teeth and metallic taste, 50% of the participants showed a positive attitude towards the use of SDF which is shown in Figure 5(see PDF). Figure 6(see PDF) depicted that majority of the participants accepted SDF treatment despite of post SDF development of or o-mucosal lesion.

## Discussion:

There is a lack of literature regarding SDF uses and its acceptance among the parent population. In regard to the gender, about 61.30% of the participants were male and 38.7% were female. Ordinarily, mothers can take the decisions about the treatment and they were more cooperative with this study. Besides, there was no statistically significant difference in acceptance ratings between male and female. There is a lack of knowledge about SDF among majority of study participants though they were aware about certain side effects and their child’s experience of the use of SDF. Majority of participants didn’t accepted SDF treatment as 58.1% of participant’s child experienced oro-mucosal lesions as they are aware of other treatment options provided in primary health care, general hospitals, or private institutions that give better aesthetic result. Although SDF has a drawback of staining teeth and metallic taste, parents show a positive attitude towards the use of SDF which is comparable to a study done by Srivastava U et al. [16]. The reason for choosing SDF may be the parent's awareness of anaesthetic agents being associated with potential risk to patient's overall health with some reports of morbidity and mortality [17-18]. Thus, SDF put forward an uncomplicated and well conventional treatment remedy to prevent active dental caries. Though parental acceptance is definitely a dilemma since it causes tooth discolouration but at the same time it can be used as an interim agent and offers an intermediate care path for paediatric population who doesn't have an immediate access to absolute treatment. Moreover SDF application may increase the quality of life of these children as perceived by parents and SDF staining is not a concern for the parents and children. Thus further studies are needed to be conducted in Indian population for parental knowledge and acceptance of SDF for their child's better oral health.

## Conclusion:

There is a lacuna in knowledge about SDF among the study participants emerging a need for proper education to them when they visit a dental setting with their child. SDF is a non invasive modality that can be chosen by parents looking for a hassle free treatment without drills which causes psychological influences to their child. It can be a boon to an uncooperative paediatric patient in a dental setting in order to reduce the progression of active caries. Although it causes discolouration of teeth post SDF treatment, still parents are willing to accept SDF as a treatment modality in the present study for their child’s oral health.
